# Feasibility of strong diffusion encoding and fast readout using a plug‐and‐play head gradient insert at 7 T

**DOI:** 10.1002/mrm.30613

**Published:** 2025-07-01

**Authors:** G. C. Arends, E. Versteeg, A. De Luca, F. Marc, T. H. M. Roos, D. W. J. Klomp, M. Froeling, J. C. W. Siero, C. M. W. Tax

**Affiliations:** ^1^ Center for Image Sciences University Medical Center Utrecht Utrecht The Netherlands; ^2^ Cardiff University Brain Research Imaging Centre (CUBRIC), School of Physics and Astronomy Cardiff University Cardiff United Kingdom; ^3^ Spinoza Centre for Neuroimaging Amsterdam Amsterdam The Netherlands

**Keywords:** diffusion MRI, gradient head insert, high field, strong gradients

## Abstract

**Purpose:**

Diffusion weighting is achieved by the application of external field gradients typically for tens of milliseconds, during which the signal also substantially decays due to inherent T_2_ relaxation. Employing strong gradients will shorten the echo time (TE) and increase the signal‐to‐noise ratio. This study demonstrates a proof‐of‐principle of using an ultrastrong head gradient insert for diffusion MRI at 7 T.

**Methods:**

A lightweight (45 kg) single‐axis gradient coil operating in the *z*‐direction was interfaced with a 7 T system and operated as an additional fourth‐gradient axis to the whole‐body gradient setup. Several diffusion MRI experiments were conducted to compare acquisitions taken with a gradient insert operating at full capacity (200 mT/m and 1300 T/m/s) to those using conventional gradient strengths (40–80 mT/m and 200 T/m/s).

**Results:**

A shorter TE of (28.8, 32.4, 38.5, 48.7) ms was achieved using the insert gradient at full capability for readout and diffusion encoding, compared to (49.7, 54.5, 65.9, 85.5) ms (80 mT/m) or (59.9, 68.8, 88.3, 120.9) ms (40 mT/m) for *b* = (0, 500, 1000, 3000, 10 000) s/mm^2^ using conventional gradient strength. A SNR increase for all *b*‐value acquisitions was observed. When using strong gradients for readout, TE was shortened by 20 ms, and a lower degree of geometrical distortions was observed.

**Conclusion:**

The results illustrate a successful proof‐of‐concept for performing diffusion MRI using a plug‐and‐play head gradient insert at 7 T. Use of low‐cost gradient inserts could make advanced diffusion MRI experiments more widely available.

## 1 INTRODUCTION

Diffusion MRI (dMRI) is sensitive to diffusion processes of water molecules and can probe the cellular microstructure of tissue in a noninvasive manner, and as such it can be used to investigate structural changes in diseases such as Alzheimer's and multiple sclerosis.^1^, ^2^, ^3^ dMRI relies on applying external magnetic field gradients for diffusion weighting (commonly characterized by the *b*‐value).^4^ Strong diffusion weighted data (i.e., *b*‐values larger than 2500 s/mm^2^) are promising to improve brain microstructure characterization.^5^, ^6^ However, with conventional hardware the diffusion encoding time of strong diffusion weighted scans becomes prohibitively long, resulting in significant signal decay due to T_2_ relaxation. The duration of the diffusion encoding for a given *b*‐value depends on the available gradient strength, which has been an incentive to build systems with stronger gradients than used in clinical MRI systems (40 mT/m).

Strong gradients offer several potential benefits: They shorten the echo time (TE), which increases the signal‐to‐noise ratio (SNR) and allows the study of compartments with short T_2_, enable faster readouts, and can probe smaller length scales. These benefits can increase the performance of dMRI in several ways and unlock new research opportunities. The ability to study short T_2_ compartments enables the investigation of myelin water.^7^, ^8^ With myelin integrity as a factor determining neurosignaling conduction speed, it may enhance our understanding of neurological disorders. Furthermore, increased gradient capabilities facilitate faster echo‐planar imaging (EPI) readouts, thus further reducing TE as well as image distortions.^9^ Stronger gradients also enable shorter diffusion times at a given *b*‐value for the study of small cellular structures such as axons^10^ and densely packed cell bodies.^11^ Finally, dMRI at high field requires shorter TE to mitigate the shorter effective T_2_. Strong gradients therefore provide an exciting prospect to revive interest in high‐field dMRI and truly benefit from its increased SNR,^12^ as well as the enhanced effect of susceptibility‐induced signal losses indicative of microstructural properties such as myelination.^13^

Strong‐gradient MRI scanners have been scarce, but recent developments in commercialization and hardware slowly increase their availability worldwide. Until now, some dedicated systems that facilitate these strong gradients have shown great potential in microstructure characterization.^14^, ^15^, ^16^, ^17^, ^18^ However, the slew rate (SR) in the whole‐body systems is limited by the risk of physiological effects such as painful peripheral nerve stimulation (PNS) and cardiac stimulation.^19^, ^20^ As such, the gain in *b*‐value is lower than the system limit (typically 200 T/m/s) would allow. Recently, dedicated systems with built‐in gradients and a smaller field of view (FOV) have shown the advantages of strong gradients for diffusion both at clinical field strengths and 7 T.^15^, ^16^, ^18^ Alternatively, plug‐and‐play gradient inserts can be considered to enhance gradient performance and make dedicated experiments for microstructural characterization more widely available at lower costs. Plug‐and‐play gradients refer to insert gradients that can be installed in existing MRI systems in under an hour. Furthermore, close‐fitting gradient coils can provide strong gradient fields that can rapidly be switched in polarity with reduced PNS. Thus, inserts can expand the functionality of current MRI systems and bridge the gap between conventional and strong gradient imaging technologies.

In this work, we investigate the feasibility of using a single‐axis plug‐and‐play gradient insert—previously developed for silent MRI readout^21^, ^22^, ^23^—for dMRI at 7 T. Due to the single‐axis (*z*‐direction) nature of the gradient insert, it has several advantages over the *x*‐ and *y*‐axes gradients, such as intrinsic force balance, high efficiency, and simple design.^24^ The implementation of a three‐axis gradient is currently in progress.^25^ We build on concepts introduced in Refs. 26, 27, 28, where the first plug‐and‐play insert gradient for dMRI was implemented. In this work, we demonstrate the feasibility of using plug‐and‐play gradient inserts at high field strength (7 T), capable of handling the high current and duty cycle required for advanced dMRI experiments. Two experiments were conducted. One aimed to demonstrate how the gradient insert can increase the SNR by shortening diffusion gradient pulses. The other aimed to utilize fast readout for high‐resolution and less distorted diffusion tensor imaging (DTI). These experiments lay the groundwork for the advancement of state‐of‐the‐art dMRI techniques with plug‐and‐play gradient inserts.

## 2 METHODS

The methods are structured as follows. The first section describes the specifications of experimental setup. The second section describes the data acquisition of the two experiments conducted in this study: Experiment 1 uses the gradient insert for diffusion weighting and readout, whereas the experiment 2 uses the gradient insert only for readout, and diffusion encoding is done by the gradients of the scanner. The last section describes the processing pipelines for both experiments.

### 2.1 Experimental setup

#### 2.1.1 Hardware

**FIGURE 1:**
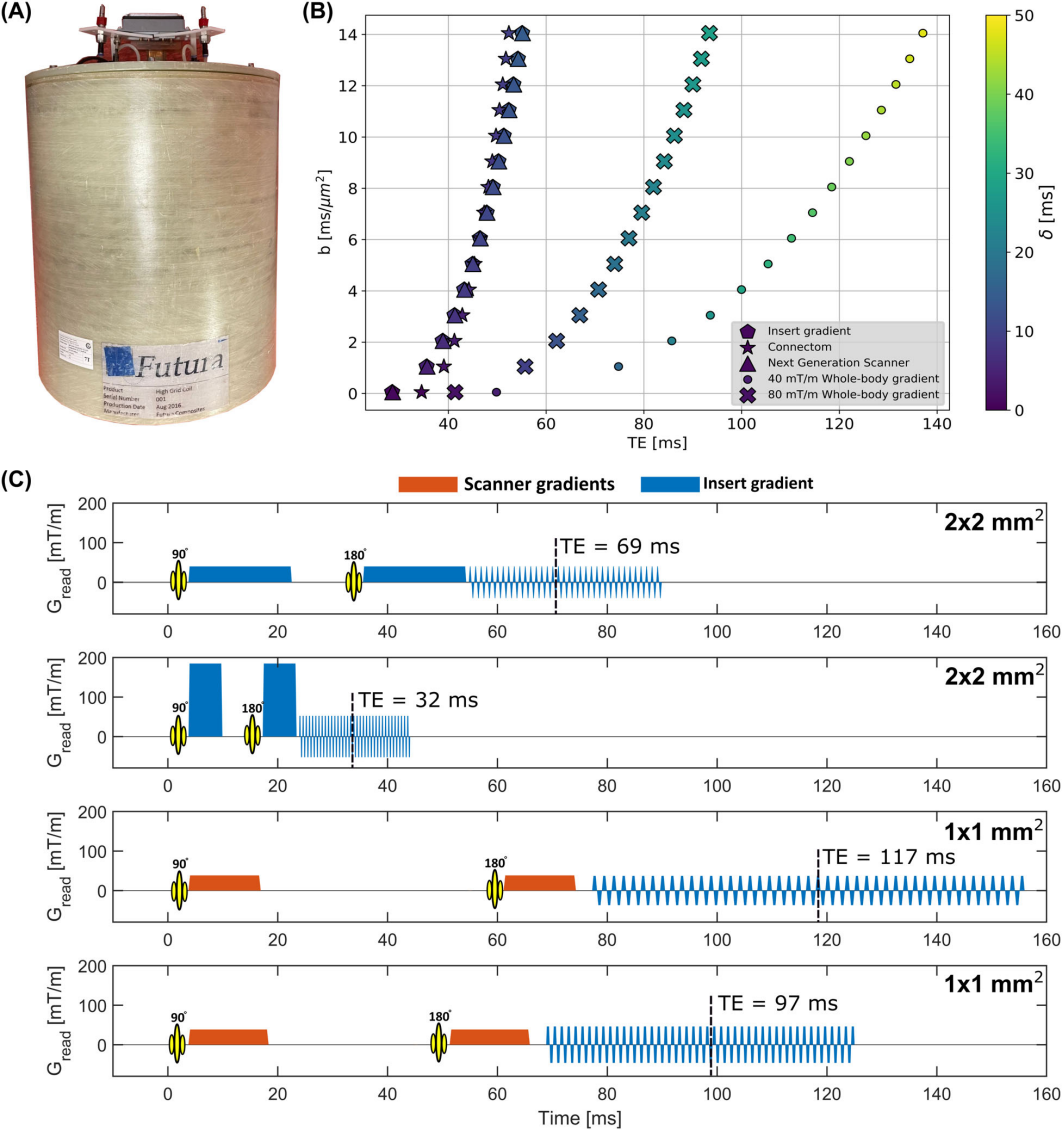
(A) Photograph of the head gradient insert.^22^ (B) Maximum achievable *b*‐value as a function of TE for different dedicated high‐performance gradient systems (300 mT/m Connectom^14^ (Siemens, Erlangen, Germany) and 200 mT/m Next Generation system^15^ (Siemens, Erlangen, Germany)) and conventional 80–40 mT/m whole‐body gradients. The readout time is assumed to be equal between systems and set to 10 ms for the high‐performance systems and 17.5 ms for the whole‐body gradient. *δ* is the length of the diffusion‐encoding gradient pulses. (C) Pulse sequences used in both experiments. The gradients are color‐coded: blue = gradient insert, orange = scanner gradients. The top two sequences are used to acquire data for experiment 1; the bottom two sequences for experiment 2. All sequences result in a diffusion weighting of *b* = 1000 s/mm^2^. The in‐plane resolution and TE are indicated in the plots

The gradient head insert consisted of a lightweight (45 kg) single‐axis gradient coil operating in the *z*‐direction (Figure 1A) (Futura, Heerhugowaard, The Netherlands),^22^ which was powered by a dedicated amplifier (1000A/1035V Prodrive Technologies, Eindhoven, The Netherlands). This combination yielded a maximum gradient amplitude and SR of 200 mT/m and 1300 T/m/s, respectively. An example of the achievable *b*‐value for a given TE compared to other strong gradient systems is shown in Figure 1B. The coil was designed for brain imaging and features a 16 cm linear region, defined based on a maximum allowable deviation of 5%, which can be corrected as described in previous studies.^17^, ^29^ It features a built‐in two‐channel RF‐transmit coil (birdcage) and could be fitted with a separate 32‐channel receive array (Nova Medical, Wilmington, MA). The inner diameter of the gradient insert is 28 cm, including the RF‐transmit coil (33 cm without the RF transmit). The gradient insert was operated as an additional fourth gradient to the whole‐body gradient setup and controlled via a dedicated gradient waveform generator (33500B, Keysight, Santa Rose, CA). To minimize forces and torques on the cable assembly, the power cables were twisted and positioned along the magnetic field.

#### 2.1.2 Standardized protocol for installation

The time, personnel, and tools required for installation have been compiled into a standardized installation protocol. A flow diagram of the protocol is shown in Figure S1, and the full protocol is available upon request. To assess the feasibility of the protocol, seven inexperienced users were asked to execute the procedure while being timed.

#### 2.1.3 Gradient impulse response function measurements

The gradient impulse response function (GIRF) was determined by applying triangular waveforms on the insert gradient and measuring the output using a dynamic field camera system (Skope, Zurich, Switzerland). The GIRF was subsequently calculated with a linear time invariant model.^30^, ^31^ A raised cosine filter was applied to suppress resonances with high frequencies not relevant for gradient waveforms. The GIRF was used for preemphasis (on diffusion gradients and EPI) and to mitigate the effect of imperfect gradient waveforms induced by eddy currents and mechanical resonances in experiment 1. An additional high‐pass filter (20 kHz) was applied to the waveforms to suppress high‐frequency resonances. Eddy currents that were not captured by the GIRF measurements were corrected in hindsight using the field camera. These k‐space readout trajectories are shown in the Figure S2.

#### 2.1.4 Acquisitions

Two healthy controls were scanned on a 7T MRI system (Philips, Best, The Netherlands) with the gradient head insert. Informed consent was given by the subject in accordance with the Institutional Review Board of the University Medical Center Utrecht. The gradient‐insert capabilities were leveraged to acquire images with strong diffusion weightings and high resolutions. Typical sequence diagrams for both experiments are depicted in Figure 1C. All scans were acquired coronally with 20 slices, with the readout from feet to head and phase‐encoding from right to left. Full capability of the insert (*G* = 200 mT/m, SR = 1300 T/m/s) is referred to as the *insert gradient mode*. Limited capability (*G* = 40 (80) mT/m, SR = 200 T/m/s) to mimic conventional gradients is called the *whole‐body gradient mode* 40 mT/m (80 mT/m). For clarification on sequence execution by the insert and scanner gradients, see the color coding in Figure 1C.

#### 2.1.5 Experiment 1: Strong diffusion weighting

dMRI scans were acquired with a pulsed gradient spin echo EPI sequence using the gradient insert mode and the whole‐body gradient mode. *b*‐values were set to *b* = (0, 500, 1000, 3000, 10,000) s/mm^2^, with TE = (28.8, 32.4, 38.5, 48.7) ms for the insert gradient mode and TE = (49.7, 54.5, 65.9, 85.5) ms (80 mT/m) or (59.9, 68.8, 88.3, 120.9) ms (40 mT/m) for the whole‐body mode. Other scan parameters were: TR = 10 s, FOV = 224 × 176 mm^2^, in‐plane resolution of 2 mm^2^, slice thickness of 2 mm, no SENSE acceleration, and partial Fourier of 0.8. Reversed phase‐encoding *b* = 0 s/mm^2^ images were acquired for all datasets. Fat suppression (spectral presaturation with inversion recovery)^32^ was used. The readout trajectories were measured using the field camera. Acoustic noise measurements were performed for *b* = (0, 500, 10,000) s/mm^2^ images using a microphone (type ecm8000, Behringer, Willich, Germany) placed in a position mimicking the location of a subject's ear. The audio waveforms were recorded and processed in MatLab (R2021a) (MathWorks, Natick, MA), and the setup was calibrated using a sound calibrator (type 4231, Brüel & Kjær, Naerum, Denmark).

#### 2.1.6 Experiment 2: High‐resolution imaging

*b* = 1000 s/mm^2^ scans were obtained with 16 uniformly distributed directions using the whole‐body gradients. The gradient insert was only used for the EPI readout to shorten the TE. Two scans were acquired with varying in‐plane resolution: 1 and 0.8 mm^2^. TE for both resolutions was 97 ms. Gradient strength and SR during the EPI readout were 73 mT/m and 800 T/m/s. For comparison, another scan was acquired using the insert for readout on whole‐body gradient mode (*G* = 37 mT/m, SR = 153 T/m/s) with in‐plane resolution of 1 mm^2^ and TE 117 ms. Other scan parameters were: TR = 3 s, FOV = 224 × 224 mm^2^, slice thickness of 4 mm. All scans used a SENSE factor of 2.5 in the phase‐encoding direction and were repeated with reversed phase encoding, giving a total of six scans. Fat suppression (spectral presaturation with inversion recovery) was used. Pulse sequence parameters for *b* = 1000 s/mm^2^ with regard to both experiments can be found in Table 1.

**TABLE 1:** Pulse sequence parameters of *b* = 1000 s/mm^2^ images.

Parameter	Experiment 1: whole‐body 40 mT/m	Experiment 1: whole‐body 80 mT/m	Experiment 1: insert gradient	Experiment 2: whole‐body 40 mT/m	Experiment 2: insert gradient
TE (ms)	69	54	32	117	97
Diffusion time (∆) (ms)	25	19	10	35	31
Readout time (ms)	36	37	10	79	56
EPI bandwidth (Hz/pixel)	3304	3831	4360	1577	1961
Echo spacing (ms)	0.51	0.53	0.29	0.89	0.63
Ramp sampling (ms)	0.31	0.41	0.052	0.38	0.17
EPI factor	70	70	70	89	89
RMS current (A)	41	55	91	–	–

*Note*: RMS current of experiment 2 are not given because they are mainly played out on the scanner gradients.

### 2.2 Processing

#### 2.2.1 Experiment 1: Strong diffusion weighting

Reconstruction was performed offline in MatLab (R2021a) (Mathworks) using a conjugate gradientSENSE^33^ algorithm, measured readout trajectories, and a nonuniform Fourier transform (gpuNUFFT^34^). After reconstruction, the data were corrected for susceptibility distortions with the reversed phase‐encoding *b* = 0 s/mm^2^ image^35^ and the propagation of gradient nonuniformities in the B‐matrix.^36^ The spatially varying gradient nonuniformities of the insert gradient were calculated with the coil configuration of the gradient insert and the Biot–Savart law. The apparent diffusion coefficient (ADC) was estimated with the *b* = 0 s/mm^2^ and *b* = 1000 s/mm^2^ images. SNR estimation was performed by calculating the mean and SD of a 5 × 5 × 2 voxels region of interest in the top right part of the corona radiata of the unprocessed *b* = 0 s/mm^2^ images (also for experiment 2). Finally, global intensity variations were corrected using an estimated low spatial frequency bias field on the *b* = 0 s/mm^2^ images.

#### 2.2.2 Experiment 2: High‐resolution imaging

The reconstruction method was similar to experiment 1. Theoretical readout trajectories were used. The preprocess consisted of Marchenko‐Pastur Principal Component Analysis denoising,^37^ Gibbs deranging,^38^ and correction for eddy currents/motion/susceptibility distortion with FMRIB Software Library^39^ using reversed phase‐encoding scans. The diffusion tensor was estimated with weighted linear least squares^40^ using the ExploreDTI^41^ software package (version 4.8.6) and B‐matrix provided by the scanner.^36^

## 3 RESULTS

### 3.1 GIRF results

**FIGURE 2:**
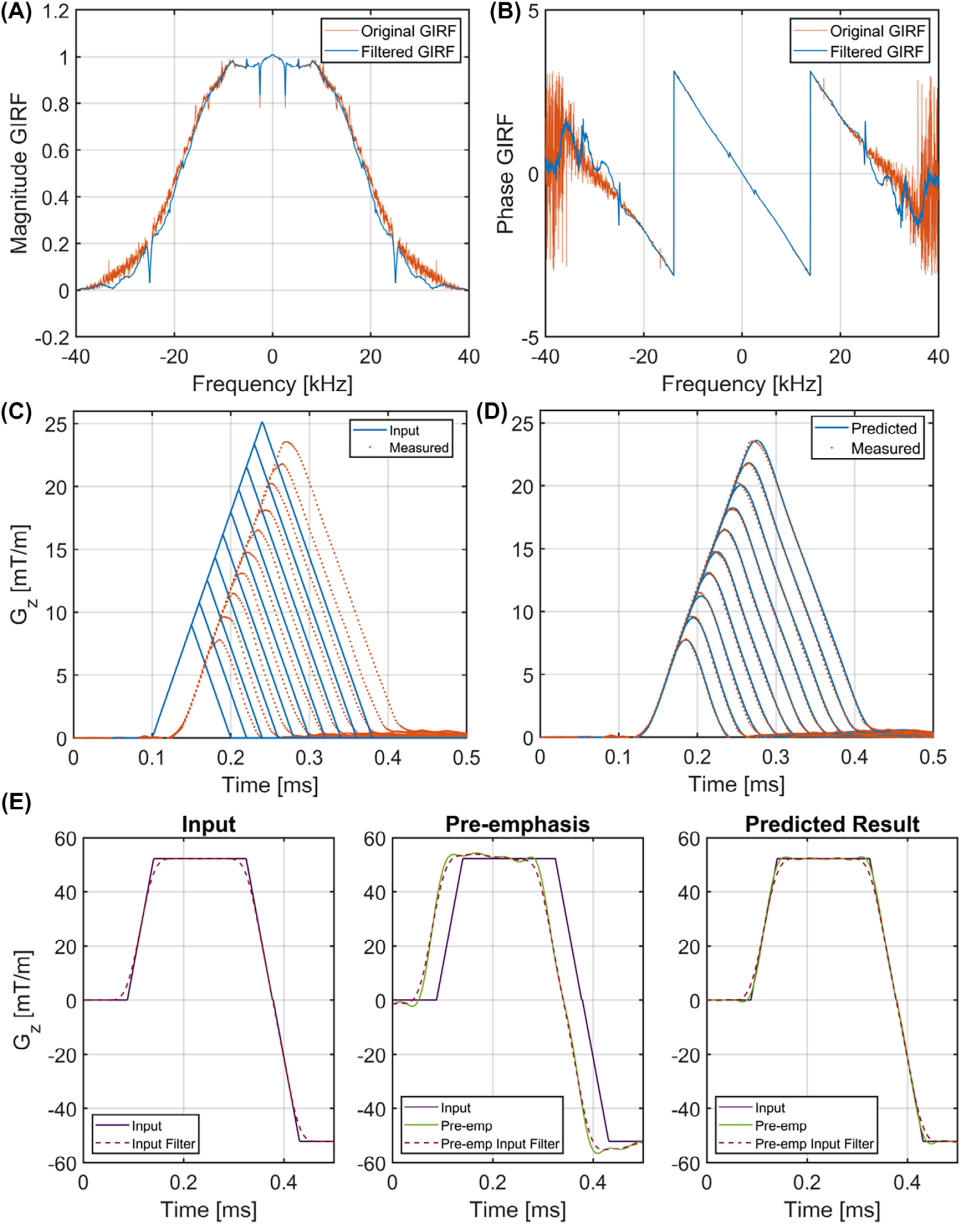
Overview of GIRF measurements and impact of preemphasis. (A) Magnitude of the GIRF with and without a raised cosine filter. (B) Phase of the GIRF. (C) Input gradient triangles and measured triangles using a field camera. (D) Measured gradient triangles and predicted wave forms using the GIRF. (E) Influence of the GIRF on EPI readout trapezoids with preemphasis and waveform 20 kHz filter GIRF, gradient impulse response function.

The GIRF (see Figure 2A,B) was successfully measured using 10 triangle waveforms (see Figure 2C,D) with gradient strengths up to 25 mT/m. Predicted waveforms by the linear time‐invarient model show good agreement with the measured output using the field camera. Figure 2E shows the effect that GIRF preemphasis has on the EPI readout gradient waveform.

### 3.2 Standardized protocol

Tests with seven inexperienced users following a standardized protocol demonstrated that the gradient insert was easy to install without requiring familiarity with complex tools. The average installation time, including the field camera setup, was 75 min. Experienced users could install the gradient insert within 15 min. Participants did not encounter significant difficulties during the process.

### 3.3 Experiment 1: Strong diffusion weighting

**FIGURE 3:**
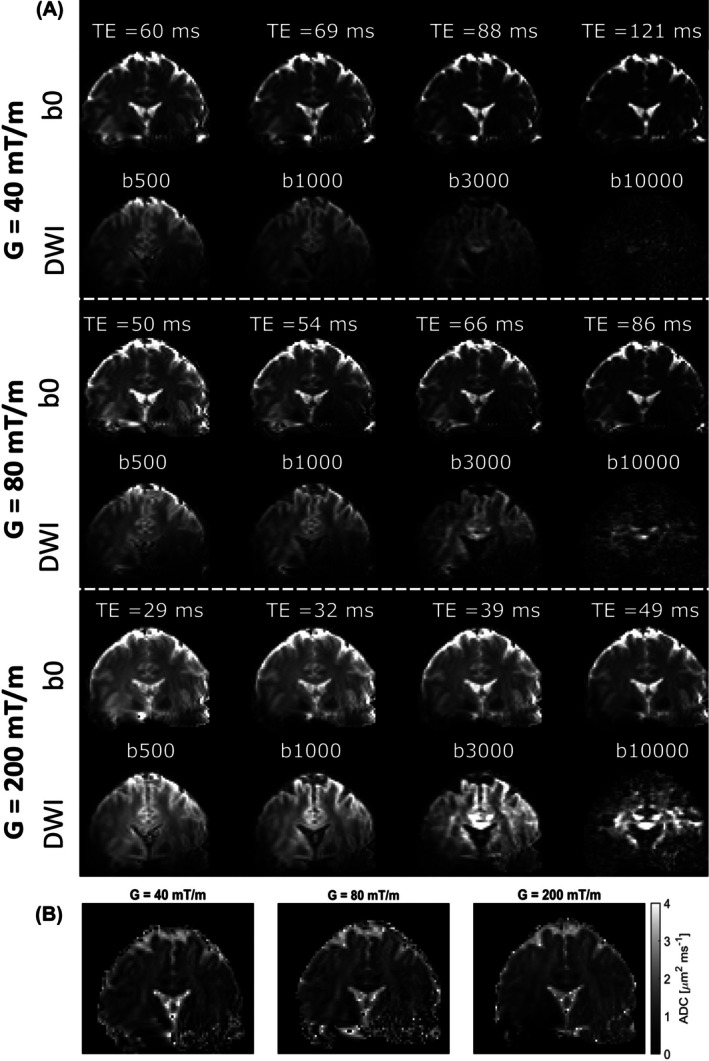
Overview of the strong diffusion weighting experiments. (A) dMRI scans with corresponding TE. The top two rows are acquired with whole‐body gradient mode; the bottom two rows with insert gradient mode. dMRI scans are scaled per *b*‐value. All *b* = 0 s/mm^2^ scans are scaled between 0 and 150. The diffusion weighted scans are scaled to the maximum of 80 mT/m whole‐body acquisition. The corresponding *b*‐value for each scan is indicated above the image. (B) ADC estimated from the *b* = 1000 s/mm^2^ and *b* = 0 s/mm^2^ scans dMRI, diffusion MRI.

**FIGURE 4:**
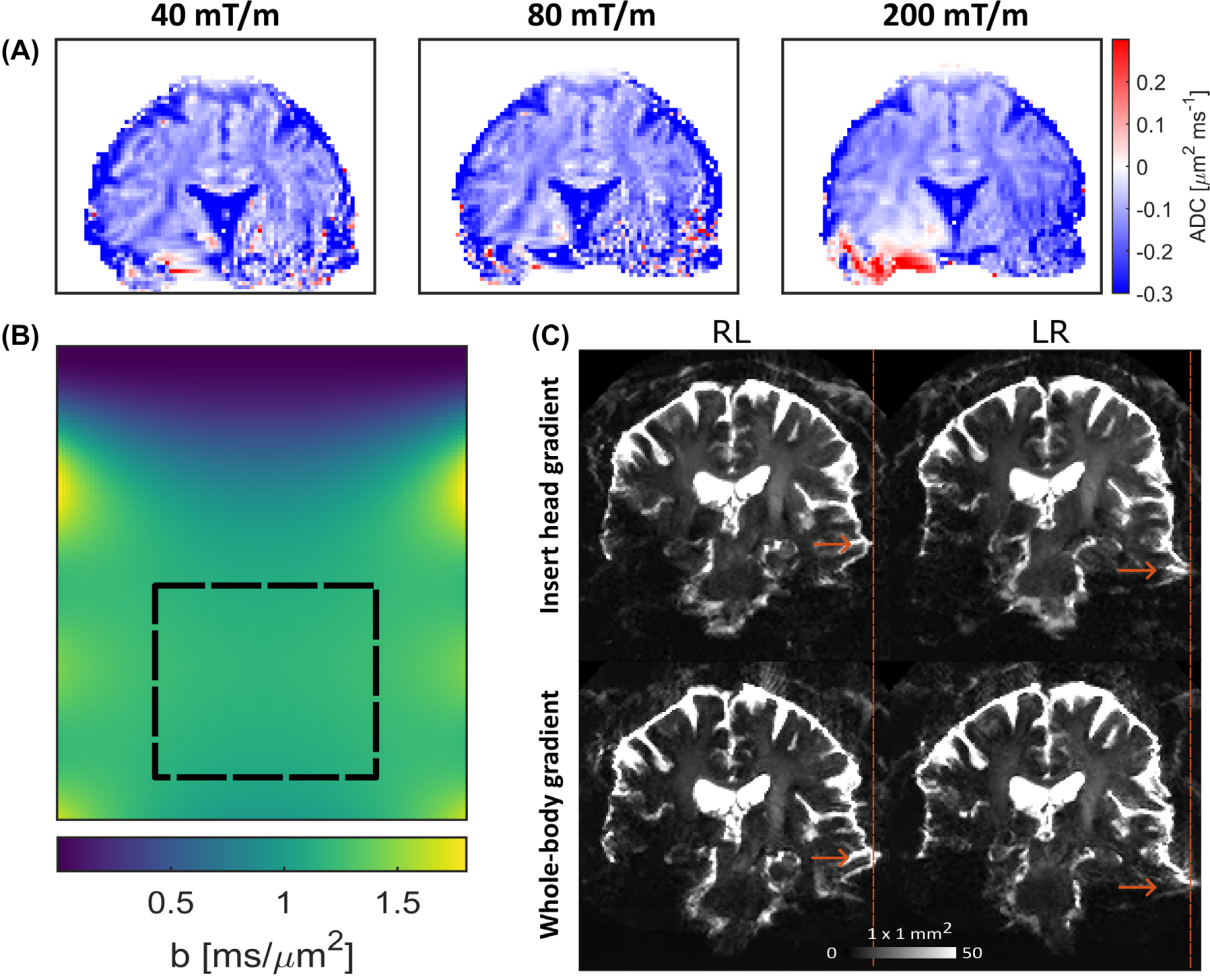
Artifact mitigation. (A) Influence of gradient nonuniformity on ADC estimation. (B) The effective *b*‐value map of *b* = 1000 s/mm^2^ of the gradient insert that was used for the gradient spatial nonuniformity correction. The imaging region is indicated by the black rectangle. (C) Comparison of EPI distortions between whole‐body and insert gradient acquisitions. Four *b* = 0 s/mm^2^ images are shown before correction. The direction of the frequency encoding is indicated above the figure. Arrows indicate example locations where fewer EPI distortions are observed for the insert gradient acquisition L, left; R, right.

Figure 3A shows dMRI scans acquired with whole‐body gradient mode (top rows) and with insert gradient mode (bottom). An increase in signal intensity can be observed when comparing whole‐body gradient mode data to insert gradient mode data, with the difference being most pronounced at high *b*‐values. The *b* = 0 s/mm^2^ images confirm the lower signal intensity for high TE. SNR calculations for the *b* = 0 s/mm^2^ images confirm the expected decrease with increasing TE. In insert gradient mode, the SNR for the *b* = 0 s/mm^2^ image corresponding to the *b* = 10 000 s/mm^2^ acquisition was 16.0, whereas the 80^40^ mT/m whole‐body gradient mode yielded an SNR of 10.3(6.1). All SNR values are plotted versus TE in Figure S3. Figure 3B shows ADC for the insert gradient mode and whole‐body gradient modes. Difference maps of ADC before and after correcting the B‐matrix for gradient nonuniformity in Figure 4A indicate an overestimation of ADC when correction is omitted, most significant in CSF (∼ 0.4 μm^2^/ms). This can be expected from the effective *b*‐value map of the insert gradient, which shows a higher effective *b*‐value in the imaging region. Results of acquisitions with 40 and 200 mT/m maximum gradient amplitude in a second subject can be found in Figure S4. The peak A‐weighted sound levels were measured at 109 dB (A) for the three experiments (*b* = 500 s/mm^2^ and *b* = 10 000 s/mm^2^). Analysis of the waveforms indicated that the peak sound level corresponds to the diffusion gradient rather than the EPI readout. No differences in gradient strength were observed. The peak corresponding to the EPI readout was different: 102.4 dB (A) for the whole‐body mode and 104.6 for the insert mode.

### 3.4 Experiment 2: High‐resolution imaging

**FIGURE 5:**
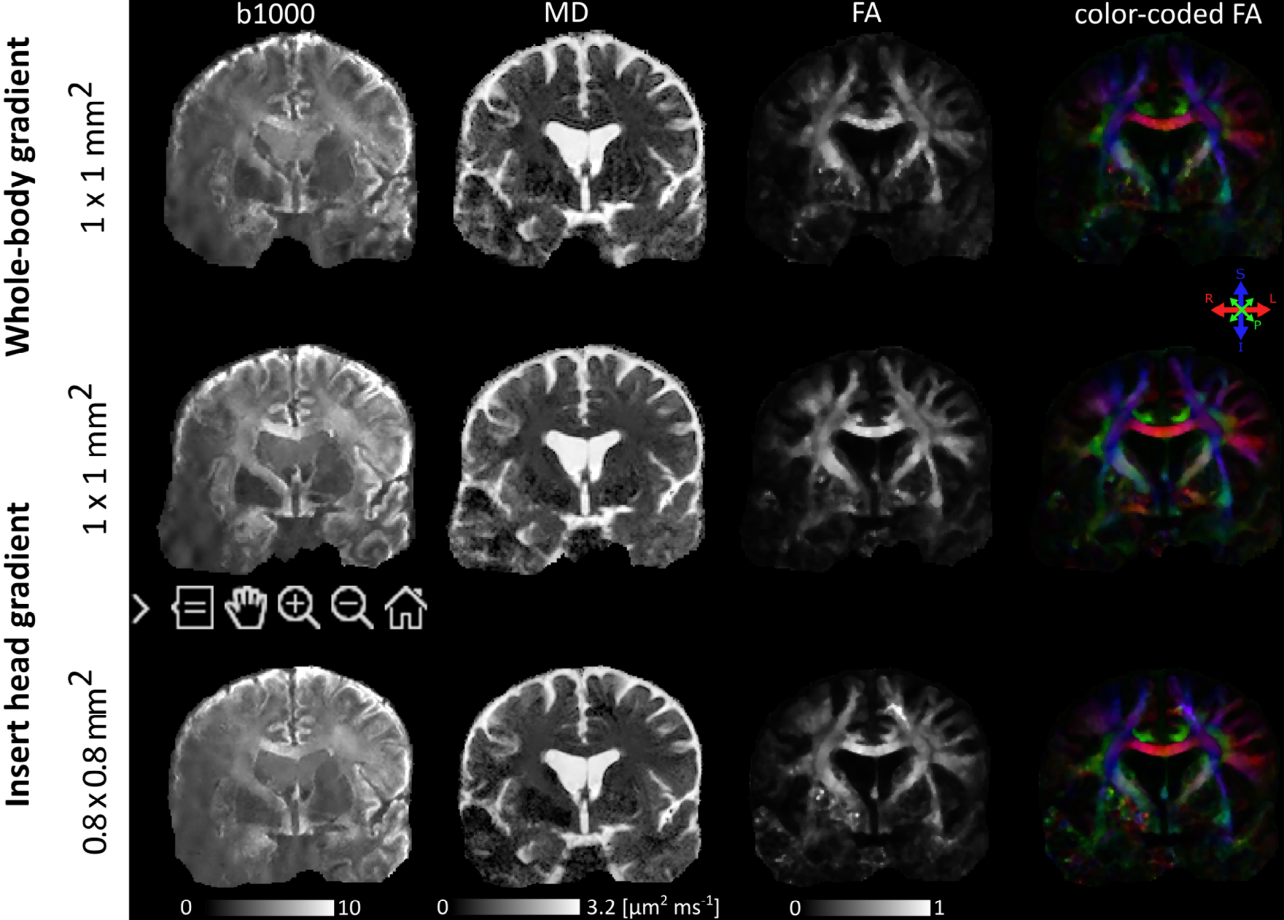
High‐resolution DTI data. The top row is 1 mm^2^ data acquired with whole‐body gradient mode for readout (TE = 117 ms). The middle and bottom rows are taken with the gradient insert mode for readout (TE = 97 ms) with in‐plane resolution 1 and 0.8 mm^2^, respectively. Every row shows a dMRI scan and mean diffusivity (MD) , FA, and color‐coded FA maps FA, fractional anisotropy.

Results of experiment 2 are presented in Figures 4C and 5. Figure 4C shows the difference in EPI distortions before correction between the whole‐body gradient mode and insert gradient mode acquisitions, with the former having more pronounced distortions. The differences can be appreciated further in the GIF added to the supplementary material (see Video S1). DTI results are presented in Figure 5. The fractional anisotropy (FA) maps show a higher contrast between the white matter and the gray matter near the cortex when comparing the 1 mm^2^ gradient insert acquisition because the whole‐body gradient results are influenced more by unrealistic high FA values in certain voxels due to low SNR. The SNR estimation, derived from a homogeneous white matter region (corona radiata) of the unprocessed *b* = 0 s/mm^2^ images, also indicates a higher SNR of 6.56 for insert gradient mode versus a SNR of 5.16 for whole‐body gradient mode. The whole‐body gradient acquisition shows Gibbs‐ringing artifacts in the frequency‐encoding direction at the border of CSF and corpus callosum in the *b* = 0 s/mm^2^ images due to a large transition in signal intensity.

## 4 DISCUSSION

The goal of this study was to show the feasibility of using a plug‐and‐play gradient insert to achieve ultrahigh *b*‐values and high resolution in dMRI. The gradient insert enabled *b*‐values up to 10,000 s/mm^2^ at short TE (49 ms). In addition, DTI scans were successfully obtained at in‐plane resolutions of 0.8 and 1.0mm^2^.

### 4.1 Operation of the gradient insert: Installation and safety

The standardized protocol enables rapid installation of the insert gradient. It can be carried out by a single person, except for lifting the insert onto the table, which requires two people to comply with local occupational health and safety regulations. The coil design ensures consistent positioning in the superoinferior and anteroposterior directions, preventing misalignment. The coil is held in place by a foam holder that can be integrated in the MRI table and has a similar curvature as the insert gradient. Small perturbations in the foot–head direction do not apply torque to the coil when placed in the isocenter. Additionally, the coil is not operated far outside the isocenter of the magnet (>30 cm), further ensuring mechanical stability. The acoustic noise measurements indicate that peak noise levels can be mitigated with adequate hearing protection for the experiments included in this study. PNS threshold characterizations for the gradient insert used here can be found in Ref. 22.

### 4.2 EPI distortions

One main advantage of using a gradient insert is the fast readout that reduces EPI‐induced geometrical distortions (see Figure 4C). These observations are consistent with previous research, which reports that EPI distortions are reduced when higher SR values and stronger gradient strengths are employed during readout.^9^, ^14^ These distortions can be reduced even further by combining a high‐performance gradient with parallel imaging.^42^ The gradient insert mitigates distortions by means of shorter attainable EPI echo spacing,^22^ increasing the phase‐encoding bandwidth. Shorter EPI echo spacing is possible due to the increased gradient strength and slew‐rate, with higher PNS limits enabled by the compact gradient field extent of the head insert. Additionally, the use of a separate (more powerful) gradient amplifier enables gradient strengths higher than conventional whole‐body gradients, effectively further shortening the readout and TE. Remaining distortions are efficiently corrected by using a reversed phase‐encoding *b* = 0 s/mm^2^ scan. However, it should be noted that the fast EPI readout comes with a trade‐off because the required higher receiver bandwidth results in lower SNR. We find that this SNR penalty is exceeded by the gained SNR due to less T_2_ decay and the mitigation of EPI distortions.

### 4.3 Gibbs‐ringing artifacts

The Gibbs‐ringing artifacts in the whole‐body gradient acquisition were addressed using a total variation filter^43^ and subvoxel shifting methods.^38^ However, these methods could not fully correct the observed Gibbs ringing in the high‐resolution whole‐body mode data, likely because the ringing signal in the brain tissue was approximately as large as the white matter signal due to the low SNR. Consequently, this results in unrealistic FA values in the corpus callosum for the whole‐body gradient data. Subvoxel shifting methods were able to correct for Gibbs ringing in the insert gradient data because the tissue signal intensity is higher. This highlights the impact of the gradient insert in enabling shorter TEs and a higher SNR.

### 4.4 Gradient nonuniformities

Results in Figure 4A,B indicate that diffusion coefficient values are affected substantially by gradient spatial nonuniformity. The necessity of gradient nonuniformity correction for accurate ADC estimation is in agreement with previous studies that investigated the influence of gradient nonuniformities on diffusion parameters.^44^, ^45^ We propose that this correction is particularly important for high‐performance gradients because stronger gradient fields can lead to larger deviations.

### 4.5 SNR improvements

Overall, the results in this study indicate higher SNR for both experiments when data was acquired with the gradient insert. This was expected because the gradient insert scans were all acquired with a shorter TE compared to using whole‐body gradients. Other methods could be used to characterize SNR, such as noise estimation from the background or MPPCA, but this requires multiple dMRI images (one gradient direction was applied in experiment 1).

### 4.6 Limitations and future work

In macroscopically or microscopically anisotropic tissue such as brain white matter, gradient direction should be varied to achieve a more complete picture of tissue microstructure. The current work shows a proof of principle with a single‐axis plug‐and‐play head gradient insert at 7 T, and future work will focus on implementation of a head gradient insert with multiple axes to match gradient strengths of available dedicated head gradients.^15^, ^16^, ^17^ This will allow for faster diffusion encoding and readout in all directions. By leveraging strong gradients both for diffusion encoding and image readout in any direction, high‐resolution dMRI (e.g., the 0.8 mm^2^ acquisition in Figure 5) with higher SNR can be achieved. The availability of diffusion encoding in three directions provides possibilities for using the gradient insert to study non‐Gaussian diffusion properties in anisotropic tissue, for example, with diffusional kurtosis imaging^46^ and biophysical models. One particular promising application of high‐performance gradient inserts is the study of biophysical models using oscillating gradient spin‐echo sequences,^47^ for example, POMACE^48^ and IMPULSED.^49^ Here, the strong gradient strength and high SR slew rate of a gradient insert can achieve frequencies and *b*‐values that up to now were only available in animal studies or in studies with dedicated MRI systems with built‐in head inserts.^50^, ^51^ Using the insert gradient mode makes it possible to achieve a *b*‐value of *b* = 400 s/mm^2^ at a frequency of 100 Hz with a gap‐filled cosinoidal waveform: three periods and ∆ = 32 ms.^52^

Image quality can be further improved by decreasing the signal loss in the temporal lobes due to $B_{1}^{+}$ inhomogeneities. These $B_{1}^{+}$ inhomogeneities originate from the nonshielded quadrature transmit coil currently used for the head insert. They could be alleviated by replacing the current transmit coil by a multi‐transmit array^53^ or by incorporating an RF shield that is transparent to the strong gradient SR values. The image readout can also be reduced by using SENSE acceleration or adopting alternative strategies such as spiral readout.^7^, ^54^ Alternatively, the gradient enables ultrasonic switching, which can be used during the readout. A previous study with the gradient insert has demonstrated the possibility of doing silent spatial encoding,^21^ which would allow accelerated EPI readout and greatly enhance patient comfort during scanning. For diffusion sequences, however, this would require combining high‐frequency spatial encoding with diffusion gradients, which is ongoing work.^55^

Together, the results show great promise for extending the use of gradient inserts for dMRI at 7 T. Gradient inserts can contribute to dMRI by improving SNR at 7 T, as demonstrated by simulations (see Figure S5), and by leveraging the enhanced effect of increased susceptibility‐induced signal losses that reveal crucial microstructural properties.^13^ Furthermore, gradient inserts have the potential to make strong diffusion encoding experiments more accessible because of the plug‐and‐play nature of the experimental setup. Due to these advantages, we envision further development of gradient inserts in the future. Several efforts have already been made outside the brain,^56^, ^57^ and more work is needed to efficiently implement gradient inserts in research and in the clinic.

## 5 CONCLUSIONS

This study has shown a proof‐of‐concept of ultrastrong diffusion encoding in the *z*‐direction and short EPI readout with a plug‐and‐play gradient head insert at 7 T. Compared to whole‐body gradients, the insert enables lower TEs and higher spatial resolution up to 0.8 mm^2^ with acceptable SNR. Plug‐and‐play inserts show great promise to support the wider implementation of strong diffusion encoding experiments.

## FUNDING INFORMATION

c.m.w.t. is supported by a Vidi grant (21299) from the Dutch Research Council (NWO), a Sir Henry Wellcome Fellowship (215944/Z/19/Z), Health Holland Scan2GO, and Eurostars FEM scan. j.c.w.s. is supported by the Brain Center Young Talent Fellowship 2019 of the University Medical Center Utrecht, The Netherlands.

## Supporting information


**Figure S1.** Flow diagram indicating the steps in the standardized protocol of installing the insert gradient for diffusion MRI. Protocol is categorized into four main steps with sub stepsindicated by images.
**Figure S2.** Readout trajectory of several different acquisitions measured with the field camera used to correct images of experiment 1. Trajectories show residual eddy currents after GIRF pre‐ emphasis.
**Figure S3.** SNR of white matter verus TE for experiment 1.
**Figure S4.** Overview of the strong diffusion weighting experiments with just 40mT/m and 200 200 mT/m. (aA) dMRI scans with corresponding TE. Top two rows are acquired with whole‐body gradient mode, bottom two rows with insert gradient mode. dMRI scans are scaled per *b*‐value. All *b* = 0 s/mm^2^ scans are scaled between 0 and 250. The diffusion weighted scans are scaled to the maximum of the corresponding insert gradient scans to highlight signal intensity differences. The corresponding *b*‐value for each scan is indicated above the image. (B) White matter SNR (corona radiata) of acquisitions plotted versus TE. (C, D) ADC maps extracted from the *b* = 0 s/mm^2^ and *b* = 1000 s/mm^2^ images of whole‐body gradient mode (C) and insert gradient mode (D).
**Figure S5.** (aA) Signal decay as a function of TE at 3 T (blue) and 7 T (red), with *t*_TE_ the threshold where the signals are equal. A linear increase in signal was assumed as a function of field strength and the apparent *T*_2_ was set to 50 and 77 ms at 7 T and 3 T respectively. (bB) *t*_TE_ as a function of other settings for the signal gain and *T*_2_.

**Video S1.** EPI distortions for the whole‐body mode and insert mode placed on top of each other.
