# Supplementary material for: Feasibility of strong diffusion encoding and fast readout using a plug‐and‐play head gradient insert at 7 T
Source: Magn Reson Med. 2025 Jul 1;94(5):2304–16. doi: 10.1002/mrm.30613 (PMC12393193; doi:10.1002/mrm.30613)
Supplement: Supplementary file 1 — Figure S1. Flow diagram indicating the steps in the standardized protocol of installing the insert gradient for diffusion MRI. Protocol is categorized into four main steps with sub stepsindicated by images. Figure S2. Readout trajectory of several different acquisitions measured with the field camera used to correct images of experiment 1. Trajectories show residual eddy currents after GIRF pre‐ emphasis. Figure S3. SNR of white matter verus TE for experiment 1. Figure S4. Overview of the strong diffusion weighting experiments with just 40mT/m and 200 200 mT/m. (aA) dMRI scans with corresponding TE. Top two rows are acquired with whole‐body gradient mode, bottom two rows with insert gradient mode. dMRI scans are scaled per b‐value. All b = 0 s/mm2 scans are scaled between 0 and 250. The diffusion weighted scans are scaled to the maximum of the corresponding insert gradient scans to highlight signal intensity differences. The corresponding b‐value for each scan is indicated above the image. (B) White matter SNR (corona radiata) of acquisitions plotted versus TE. (C, D) ADC maps extracted from the b = 0 s/mm2 and b = 1000 s/mm2 images of whole‐body gradient mode (C) and insert gradient mode (D). Figure S5. (aA) Signal decay as a function of TE at 3 T (blue) and 7 T (red), with t TE the threshold where the signals are equal. A linear increase in signal was assumed as a function of field strength and the apparent T 2 was set to 50 and 77 ms at 7 T and 3 T respectively. (bB) t TE as a function of other settings for the signal gain and T 2. [file MRM-94-2304-s002.docx]

#
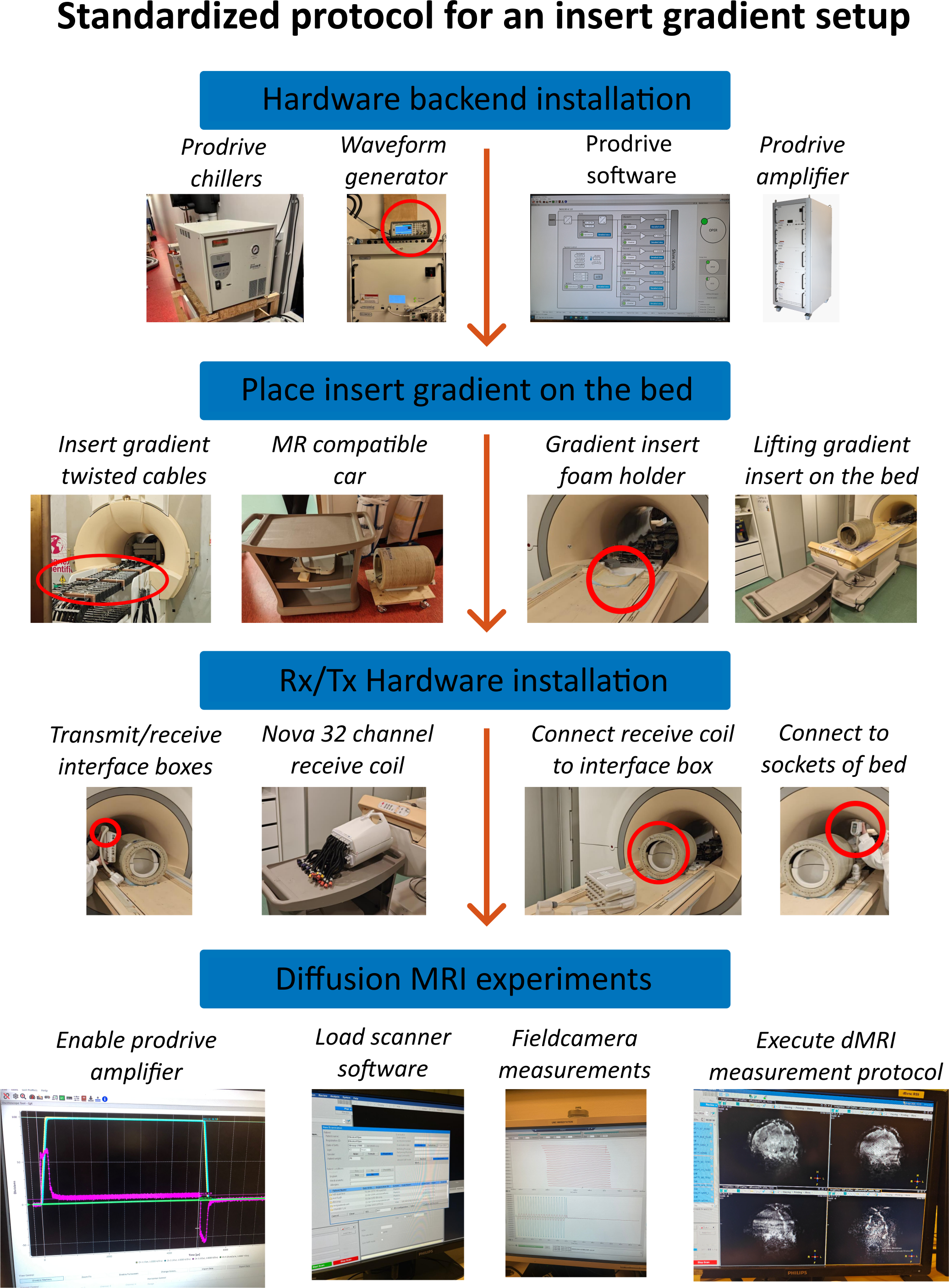
Supporting Figures and Tables

Figure S1: Flow diagram indicating the steps in the standardized protocol of installing the insert gradient for diffusion MRI. Protocol is categorized into four main steps with sub stepsindicated by images.


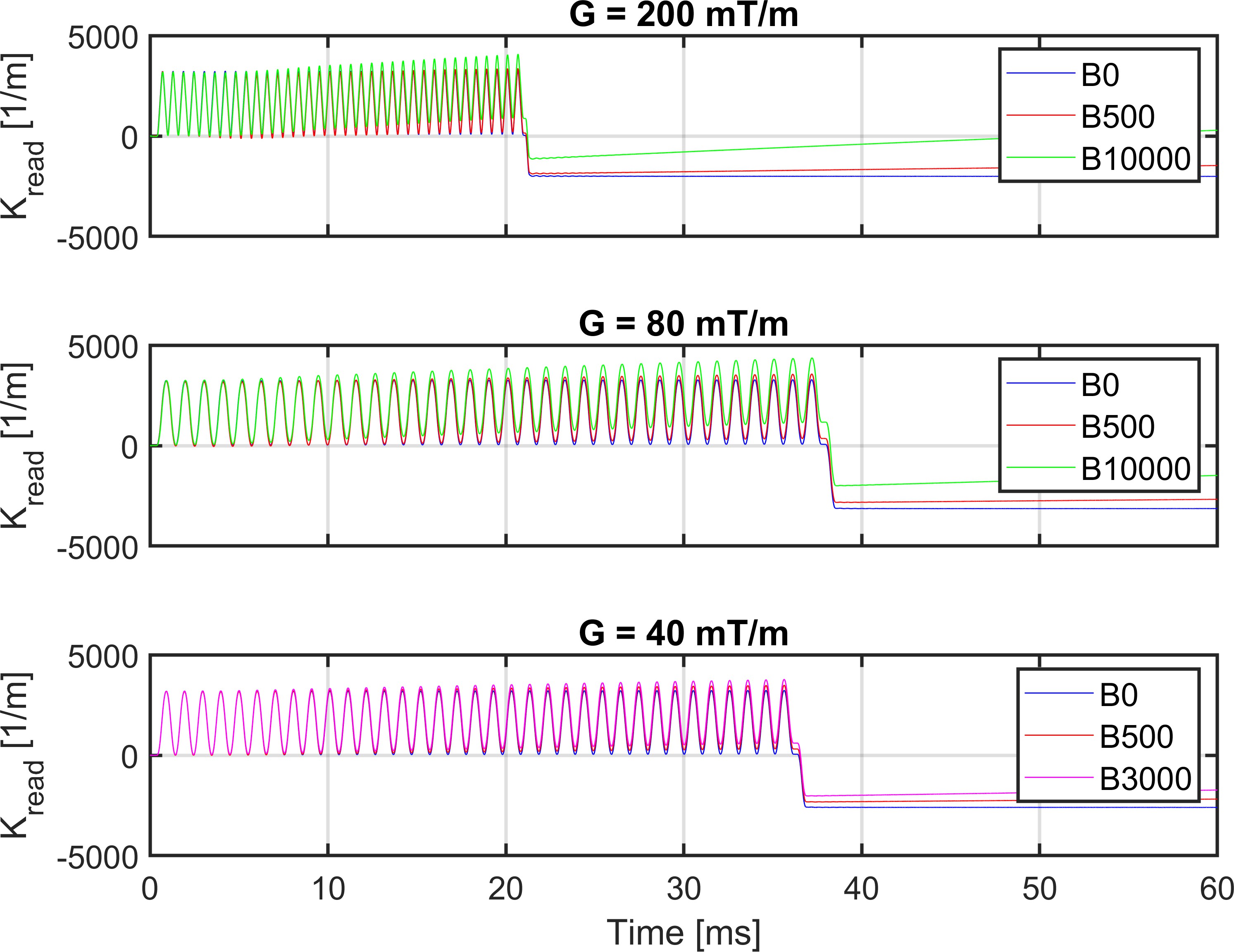


Figure S2: Readout trajectory of several different acquisitions measured with the field camera used to correct images of experiment 1. Trajectories show residual eddy currents after GIRF pre- emphasis.


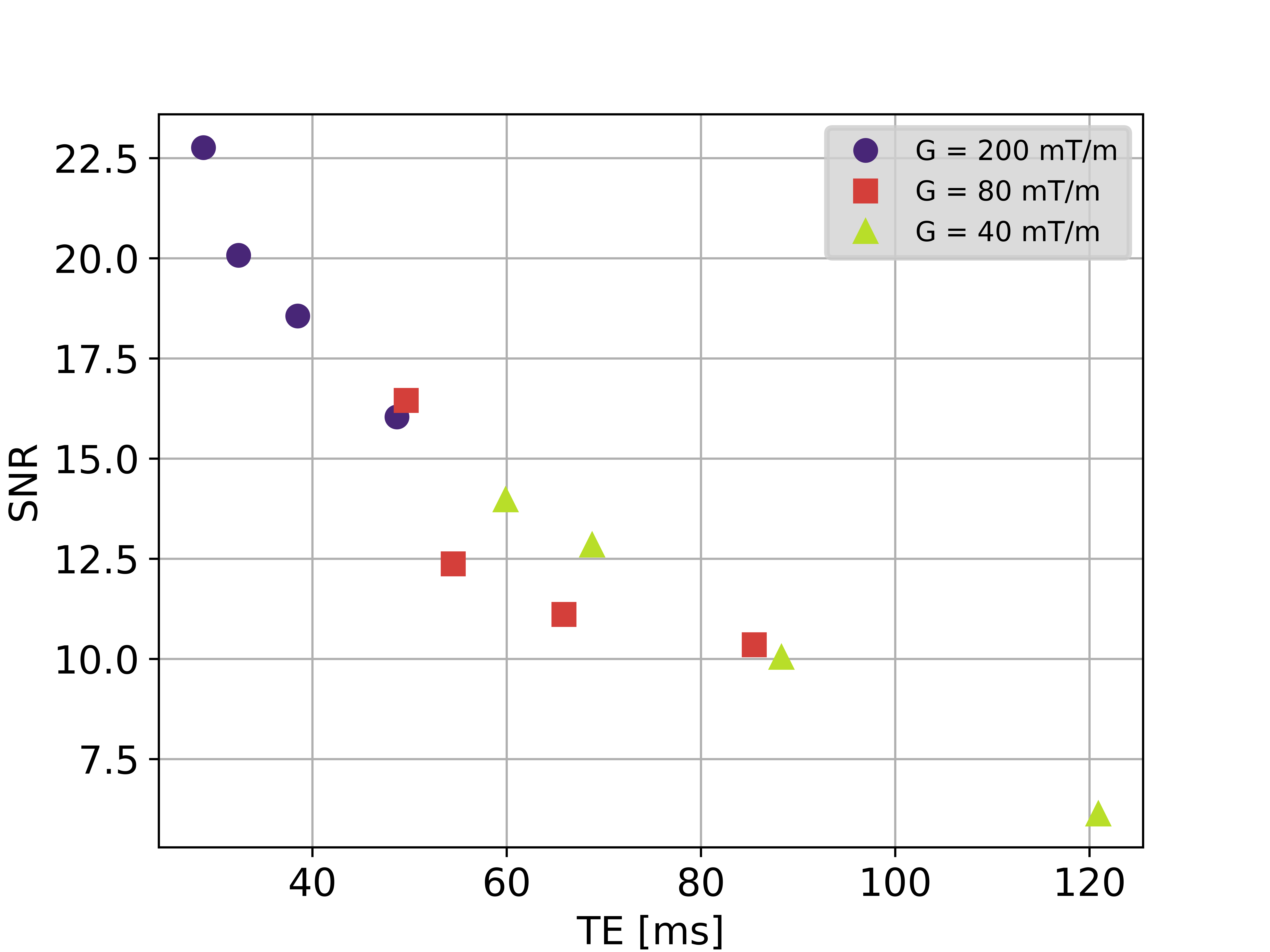


Figure S3: SNR of white matter verus TE for experiment 1.


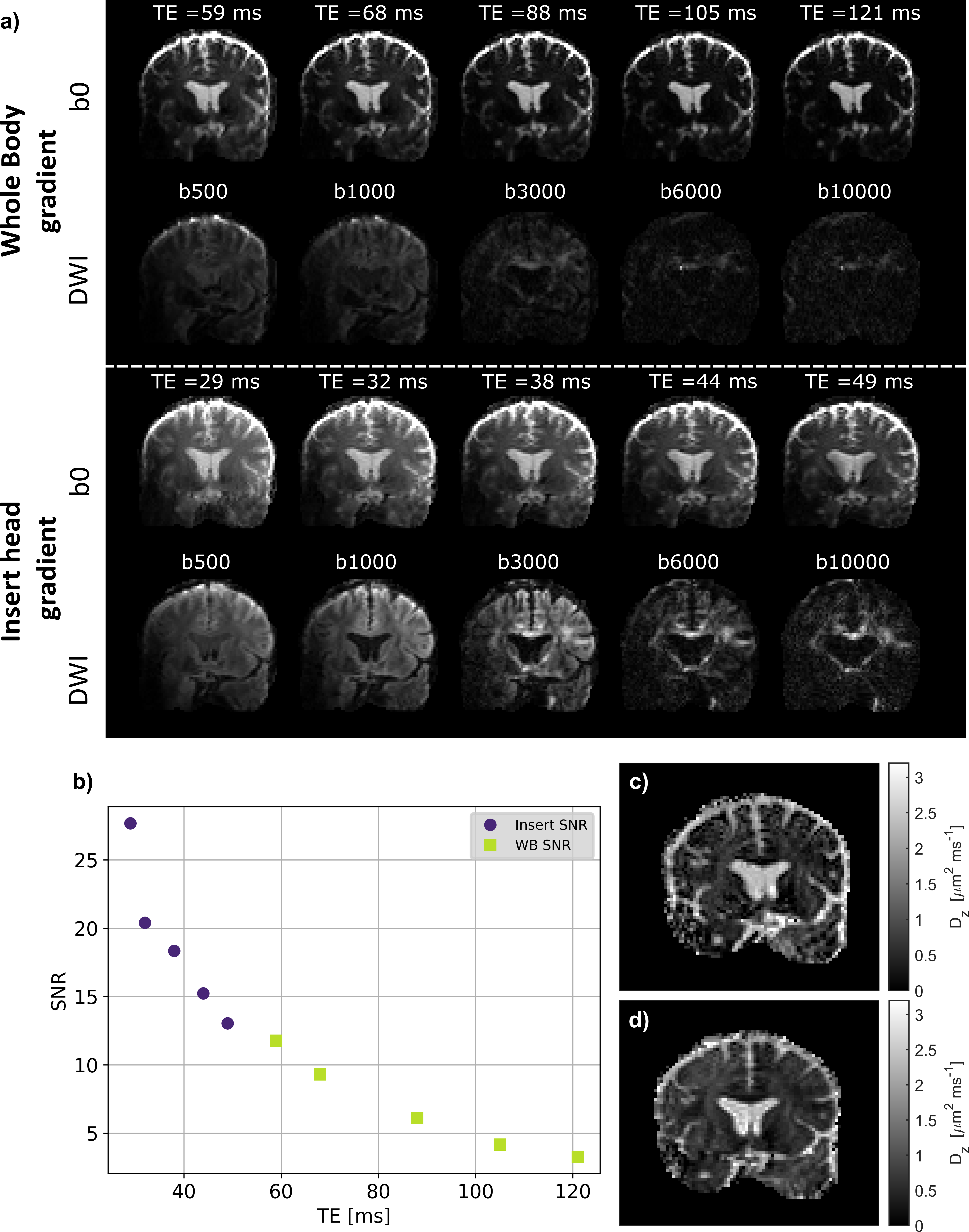


Figure S4: Overview of the strong diffusion weighting experiments with just 40mT/m and 200 mT/m. **a)** dMRI scans with corresponding TE. Top two rows are acquired with whole-body gradient mode, bottom two rows with insert gradient mode. dMRI scans are scaled per *b*-value. All *b* = 0 s/mm^2^ scans are scaled between 0 and 250. The diffusion weighted scans are scaled to the maximum of the corresponding insert gradient scans to highlight signal intensity differences. The corresponding *b*-value for each scan is indicated above the image. **b)** White matter SNR (corona radiata) of acquisitions plotted versus TE. **c,d)** ADC maps extracted from the *b* = 0 s/mm^2^ and *b* = 1000 s/mm^2^ images of whole-body gradient mode **(c)** and insert gradient mode **(d)**.


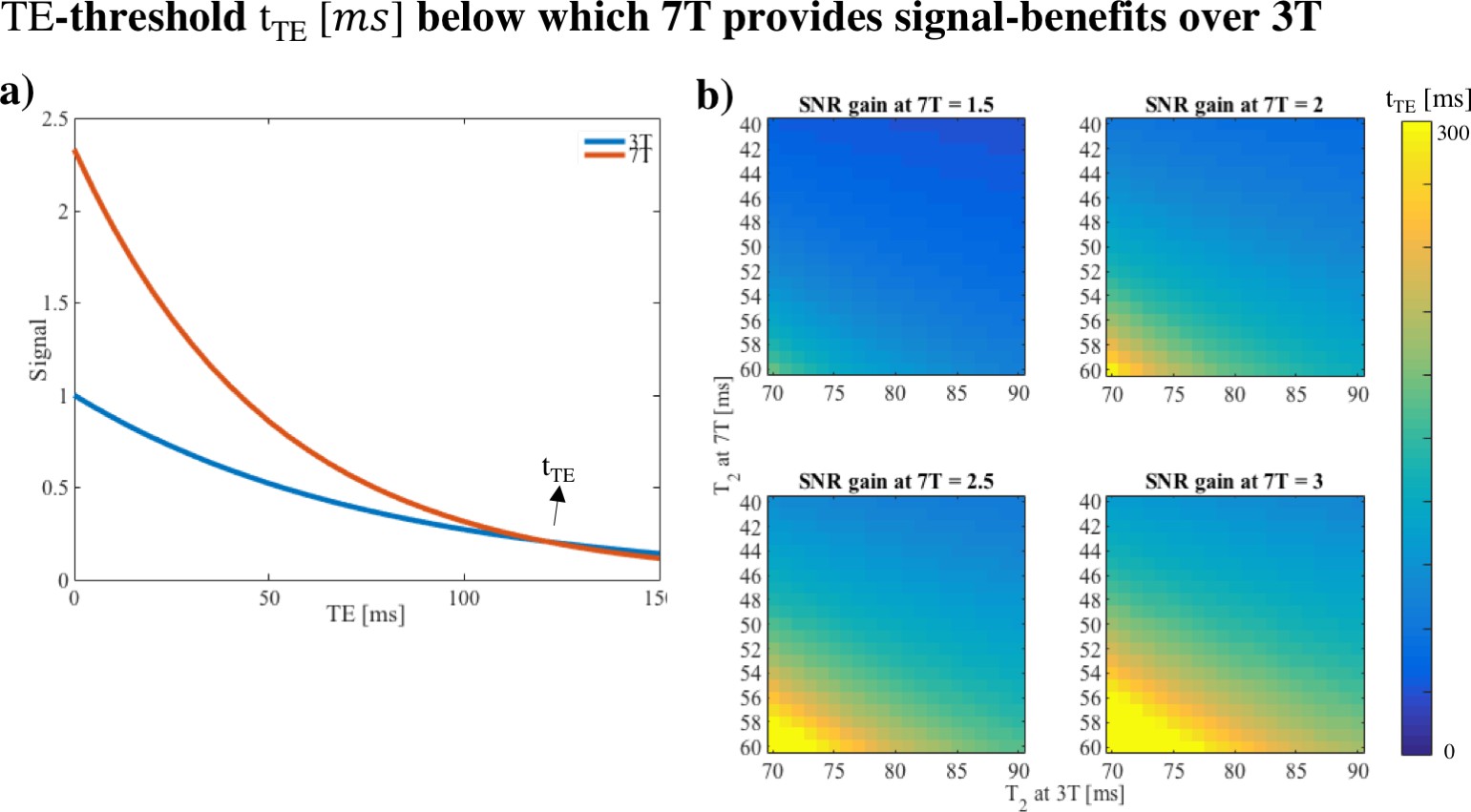


Figure S5: a) Signal decay as a function of TE at 3T (blue) and 7T (red), with *t*_TE_ the threshold where the signals are equal. A linear increase in signal was assumed as a function of field strength and the apparent *T*_2_ was set to 50 and 77 ms at 7T and 3T respectively. b) *t*_TE_ as a function of other settings for the signal gain and *T*_2_.

**Video S1**. EPI distortions for the whole-body mode and insert mode placed on top of each other.
