# Supplementary figures and images for: Feasibility of strong diffusion encoding and fast readout using a plug‐and‐play head gradient insert at 7 T
Source: Magn Reson Med. 2025 Jul 1;94(5):2304–16. doi: 10.1002/mrm.30613 (PMC12393193; doi:10.1002/mrm.30613)

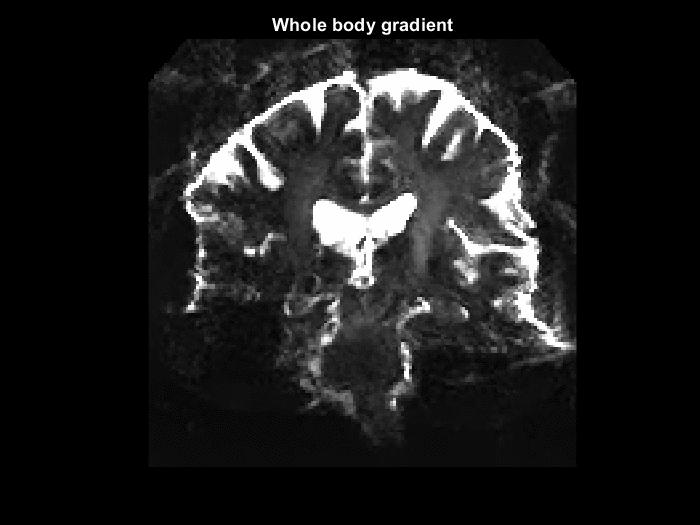

Supplement: Supplementary file 2 — Video S1. EPI distortions for the whole‐body mode and insert mode placed on top of each other. [file MRM-94-2304-s001.gif]
